# Supplementary material for: Provision of professional interpreters and Heart School attendance for foreign-born compared with native-born myocardial infarction patients in Sweden
Source: Int J Cardiol Heart Vasc. 2024 Mar 23;51:101392. doi: 10.1016/j.ijcha.2024.101392 (PMC10973978; doi:10.1016/j.ijcha.2024.101392)
Supplement: Supplementary data 1 [file mmc1.docx]

**Provision of professional interpreters and Heart School attendance for foreign-born compared with native-born myocardial infarction patients in Sweden**

**Supplement for online publication**

| **Table S1. Missing values** | |
| --- | --- |
|  | Missing n (%) |
| Age | 0 (0.0) |
| Female sex | 0 (0.0) |
| Smoking status | 39 (0.5) |
| Hypertension | 37 (0.4) |
| Diabetes melltius | 12 (0.1) |
| Previous MI | 29 (0.3) |
| Previous PCI | 38 (0.5) |
| Previous CABG | 54 (0.6) |
| Previous stroke | 22 (0.3) |
| Known LV dysfunction | 200 (2.4) |
| **Medication at admission** |  |
| Aspirin | 135 (1.6) |
| P2Y12-inhibitor | 131 (1.6) |
| ACE-inhibitor | 145 (1.7) |
| ARB | 142 (1.7) |
| Oral anticoagulation | 129 (1.5) |
| Betablocker | 153 (1.8) |
| Statin | 136 (1.6) |
| Insulin | 125 (1.5) |
| Oral antidiabetc | 130 (1.6) |
| **Socioeconomic factors** |  |
| Civic status | 4 (0) |
| Income | 4 (0) |
| Education level | 83 (1.0) |
| **Clinical findings at admission** |  |
| Systolic BP mmHg | 24 (0.3) |
| Cholesterol mmol/l | 1065 (12.7) |
| LDL-cholesterol mmol/l | 1274 (15.2) |
| GFR MDRD | 32 (0.4) |
| BMI | 251 (3.0) |
| Type of MI | 0 (0.0) |
| LV function during index | 814 (9.7) |
| **Medication at discharge** |  |
| Aspirin | 6 (0.1) |
| P2Y12-inhibitors | 1 (0.0) |
| Dual anti-platelet therapy | 4 (0.0) |
| Oral anti-coagulation | 7 (0.1) |
| ACE-inhibitors | 1 (0.0) |
| Beta-blockers | 0 (0.0) |
| Statins | 1 (0.0) |
| Insulin | 2 (0.0) |
| Oral diabetes medication | 1 (0.0) |
| Results are presented as numbers (percentages) and mean (SD). Abbreviations: MI, myocardial infarction; PCI, percutaneous coronary intervention; CABG, coronary artery bypass graft; LV, left ventricular; ACE, angiotensin converting enzyme; ARB, angiotensin receptor blocker; BP, blood pressure; eGFR, estimated glomerular filtration rate; BMI, body mass index; STEMI, ST-elevation myocardial infarction. | |

| **Table S2. Baseline characteristics** | | | | | | |
| --- | --- | --- | --- | --- | --- | --- |
|  | Foreign-born not attending Heart School N= 938 | Foreign-born attending Heart School N= 477 | P-value | Native-born not attending Heart School  N= 2990 | Native-born attending Heart School N= 3165 | P-value |
| Age (SD) | 59.3 (14) | 59.7 (14) | 0.37 | 63.4 (12) | 63.3 (12) | 0.96 |
| Female sex | 205 (21.9) | 99 (20.8) | 0.68 | 726 (22.0) | 781 (24.7) | 0.72 |
| **Smoking status** | | | | | | |
| Current smoker | 436 (46.5) | 167 (35.0) | <0.001 | 915 (30.6) | 654 (20.7) | <0.01 |
| Previous smoker | 306 (32.6) | 181 (37.2) |  | 1184 (39.6) | 1268 (40.1) |  |
| Never smoked | 196 (20.9) | 129 (27.0) |  | 915 (30.6) | 654 (20.7) |  |
| Hypertension | 421 (45.2) | 211 (44.3) | 0.78 | 1500 (50.4) | 1428 (45.3) | <0.01 |
| Diabetes mellitus | 276 (28.9) | 112 (23.5) | 0.02 | 747 (25.0) | 565 (17.9) | <0.01 |
| Previous MI | 209 (22.4) | 76 (16.0) | 0.01 | 713 (24.0) | 411 (13.0) | <0.01 |
| Previous PCI | 198 (21.2) | 69 (14.6) | <0.01 | 602 (20.2) | 384 (12.2) | <0.01 |
| Previous CABG | 69 (7.4) | 13 (2.7) | < 0.01 | 214 (7.2) | 122 (3.9) | <0.01 |
| Previous revascularization | 221 (23.8) | 74 (15.6) | < 0.01 | 385 (23.5) | 1270 (19.0) | <0.01 |
| Previous stroke | 41 (4.4) | 12 (2.5) | 0.10 | 156 (5.2) | 95 (3.0) | <0.01 |
| Known LVD | 53 (5.7) | 15 (3.2) | 0.05 | 189 (6.5) | 104 (3.4) | <0.01 |
| **Medication at admission** | | | | | | |
| Aspirin | 242 (26.4) | 98 (21.0) | 0.03 | 850 (28.9) | 610 (19.5) | < 0.01 |
| P2Y12-inhibitor | 43 (4.7) | 16 (3.4) | 0.33 | 132 (4.5) | 96 (3.1) | < 0.01 |
| ACE-inhibitor | 214 (23.3) | 72 (15.5) | <0.01 | 604 (20.6) | 526 (16.9) | < 0.01 |
| ARB | 118 (12.9) | 70 (15.0) | 0.281 | 564 (19.2) | 592 (19.0) | 0.82 |
| Oral anticoagulation | 37 (4.0) | 15 (3.2) | 0.550 | 145 (4.9) | 121 (3.9) | 0.05 |
| Betablocker | 293 (32.0) | 104 (22.4) | <0.01 | 928 (31.7) | 737 (23.7) | < 0.01 |
| Statin | 276 (30.1) | 125 (26.8) | 0.21 | 911 (31.0) | 727 (23.3) | < 0.01 |
| Insulin | 89 (9.7) | 38 (8.1) | 0.38 | 328 (11.1) | 220 (7.0) | < 0.01 |
| Oral diabetes medication | 155 (16.9) | 78 (16.7) | 1.00 | 415 (14.1) | 319 (10.2) | < 0.01 |
| **Socioeconomic factors** | | | | | | |
| Marital status |  |  |  |  |  |  |
| Married /living together | 561 (59.8) | 299 (62.8) | 0.30 | 1557 (52.1) | 2020 (63.8) | < 0.01 |
| Living alone | 377 (40.2) | 177 (37.2) |  | 1432 (47.9) | 1145 (36.2) |  |
| Income (quintiles) |  |  |  |  |  |  |
| 1 | 385 (41.0%) | 114 (23.9%) | <0.01 | 509 (17%) | 327 (10.3%) | <0.01 |
| 2 | 186 (19.8%) | 83 (17.4%) |  | 643 (21.5%) | 558 (17.6%) |  |
| 3 | 162 (17.3%) | 91 (19.1%) |  | 592 (19.8%) | 685 (21.6%) |  |
| 4 | 113 (12.0%) | 116 (24.4%) |  | 627 (21.0%) | 768 (24.3%) |  |
| 5 | 92 (9.8%) | 72(15.1%) |  | 618 (20.7%) | 827 (26.1%) |  |
| Education |  |  |  |  |  |  |
| Less than 10 years | 307 (34.7) | 107 (22.9) | <0.01 | 870 (29.1) | 747 (23.6) | <0.01 |
| 10-12 years | 370 (41.8) | 214 (45.8) |  | 1504 (50.4) | 1607 (50.8) |  |
| College/university level | 209 (23.6) | 146 (31.3) |  | 611 (20.5) | 809 (25.6) |  |
| **Clinical findings at admission** | | | | | | |
| Systolic BP mmHg (SD) | 149.4 (36) | 150.4 (34) | 0.53 | 150.3 (40) | 152.0 (35) | < 0.01 |
| Cholesterol mmol/l (SD) | 4.9 (1.7) | 5.1 (1.6) | <0.01 | 4.9 (1.7) | 5.1 (1.6) | <0.01 |
| LDL-cholesterol mmol/L (SD) | 3.0 (1.6) | 3.2 (1.6) | 0.03 | 3.0 (1.6) | 3.1 (1.5) | <0.01 |
| eGFR (MDRD) (SD) | 90.6 (30) | 91.0 (26.8) | 0.80 | 87.8 (29.8) | 88.0 (26.1) | 0.01 |
| BMI (SD) | 28.4 (5.6) | 28.1 (5.2) | 0.26 | 27.9 (5.9) | 27.9 (5.1) | 0.74 |
| STEMI | 348 (37.1) | 201 (42.1) | 0.07 | 1121 (37.5) | 1292 (40.8) | < 0.01 |
| LV function during index |  |  |  |  |  |  |
| LVEF ≥50% | 582 (68.1) | 297 (69.9) | 0.28 | 1014 (67.5) | 3932 (64.9) | 0.01 |
| LVEF = 40-49% | 156 (18.3) | 87 (20.5) |  | 293 (19.5) | 1292 (21.3) |  |
| LVEF = 30-39% | 85 (10.0) | 31 (7.3) |  | 142 (9.5) | 590 (9.7) |  |
| LVEF <30 | 30 (3.5) | 9 (2.1) |  | 51 (3.4) | 191 (3.2) |  |
| **Medication at discharge** | | | | | | |
| Aspirin | 909 (96.9) | 464 (97.3) | 0.74 | 2820 (94.4) | 3072 (97.2) | < 0.01 |
| P2Y12-inhibitors | 852 (90.8) | 444 (93.1) | 0.16 | 2735 (91.5) | 2943(93.0) | 0.32 |
| Dual anti-platelet therapy | 828 (88.3) | 433 (90.8) | 0.18 | 2600 (87.0) | 2867 (90.7) | < 0.01 |
| Oral anti-coagualtion | 73 (7.8) | 36 (7.5) | 0.92 | 297 (9.9) | 246 (7.8) | < 0.01 |
| ACE-inhibitors | 630 (67.2) | 313 (65.6) | 0.59 | 1847 (61.8) | 2015 (63.7) | 0.13 |
| ARB | 163 (17.4) | 90 (18.9) | 0.51 | 750 (25.1) | 1627 (23.0) | 0.06 |
| Beta-blockers | 854 (91.0) | 436 (91.4) | 0.84 | 2668 (89.2) | 2848 (90.0) | 0.32 |
| Statins | 903 (96.3) | 474 (99.4) | <0.01 | 2893 (96.8) | 3112 (98.4) | < 0.01 |
| Insulin | 104 (11.1) | 44 (9.2) | 0.31 | 333 (11.1) | 233 (7.4) | < 0.01 |
| Oral diabetes medication | 190 (20.3) | 81 (17.0) | 0.15 | 445 (14.9) | 357 (11.3) | < 0.01 |
| Results are presented as numbers (percentages) and mean (SD). Abbreviations: MI, myocardial infarction; PCI, percutaneous coronary intervention; CABG, coronary artery bypass graft; LV, left ventricular; ACE, angiotensin converting enzyme; ARB, angiotensin receptor blocker; BP, blood pressure; eGFR, estimated glomerular filtration rate; BMI, body mass index; STEMI, ST-elevation myocardial infarction. | | | | | | |

| **Table S3. Stop-smoking talk in patients attending Heart School compared with patients not attending Heart School** | | | | |
| --- | --- | --- | --- | --- |
|  | Attending Heart School | Not attending Heart School | P-value | Interaction  p-value |
| **Stop-smoking talk** | | | | |
| All | 201 (25.2) | 189 (14.5) | <0.01 |  |
| Foreign-born | 54 (32.9) | 62 (15.0) | <0.01 | 0.12 |
| Native-born | 147 (23.1) | 127 (14.3) | <0.01 |  |
| Results are presented as numbers (percentages). | | | | |
